# Differential responses of the gut transcriptome to plant protein diets in farmed Atlantic salmon

**DOI:** 10.1186/s12864-016-2473-0

**Published:** 2016-02-29

**Authors:** Elżbieta Król, Alex Douglas, Douglas R. Tocher, Viv O. Crampton, John R. Speakman, Christopher J. Secombes, Samuel A. M. Martin

**Affiliations:** Institute of Biological and Environmental Sciences, University of Aberdeen, Aberdeen, AB24 2TZ UK; Institute of Aquaculture, University of Stirling, Stirling, FK9 4LA UK; EWOS Innovation AS, Dirdal, Norway

**Keywords:** Soy protein concentrate, Bean protein concentrate, Soybean meal, Antinutritional factors, Gut inflammation, Enteritis, Whole-animal performance, Microarray, Gene expression, Cholesterol biosynthesis pathway

## Abstract

**Background:**

The potential for alternative plant protein sources to replace limited marine ingredients in fish feeds is important for the future of the fish farming industry. However, plant ingredients in fish feeds contain antinutritional factors (ANFs) that can promote gut inflammation (enteritis) and compromise fish health. It is unknown whether enteritis induced by plant materials with notable differences in secondary metabolism is characterised by common or distinct gene expression patterns, and how using feeds with single *vs* mixed plant proteins may affect the gut transcriptome and fish performance. We used Atlantic salmon parr to investigate the transcriptome responses of distal gut to varying dietary levels (0–45 %) of soy protein concentrate (SPC) and faba bean (*Vicia faba*) protein concentrate (BPC) following an 8-week feeding trial. Soybean meal (SBM) and fish meal (FM) were used as positive and negative controls for enteritis, respectively. Gene expression profiling was performed using a microarray platform developed and validated for Atlantic salmon.

**Results:**

Different plant protein materials (SPC, BPC and SBM) generated substantially different gut gene expression profiles, with relatively few transcriptomic alterations (genes, pathways and GO terms) common for all plant proteins used. When SPC and BPC were simultaneously included in the diet, they induced less extensive alterations of gut transcriptome than diets with either SPC or BPC singly, probably due to reduced levels of individual ANFs. The mixed plant protein diets were also associated with improved body composition of fish relative to the single plant protein diets, which may provide evidence for a link between the magnitude of changes in gut transcriptome and whole-animal performance.

**Conclusions:**

Our results indicate that gut transcriptomic profiling provides a useful tool for testing the applicability of alternative protein sources for aquaculture feeds and designing diets with reduced impact of ANFs on fish health. Ultimately, understanding diet-gut interactions and intestinal homeostasis in farmed fish is important to maximise performance and to ensure that aquaculture continues to be a sustainable source of food for a growing world population.

**Electronic supplementary material:**

The online version of this article (doi:10.1186/s12864-016-2473-0) contains supplementary material, which is available to authorized users.

## Background

The Atlantic salmon (*Salmo salar* L.) is a carnivorous species and its natural diet consists primarily of aquatic invertebrates in early life and then crustaceans and small fish during the marine phase of growth in later life. Farmed salmon were historically fed diets containing fish meal (FM) as the primary protein source to mimic their natural feeding habits [1]. With the rapid expansion of the salmon farming industry and the limited availability of wild-caught fish, the global contribution of FM to salmon feeds has decreased from ~45 % in 1995 to ~20 % in 2012 and is predicted to reduce to ~12 % by 2020 [2]. Thus, the future of salmon farming will depend to a large extent on sustainable dietary protein alternatives to FM.

In recent years, significant progress has been made towards replacing FM with plant protein meals and plant protein concentrates [3]. The large-scale evaluation of plant protein sources for aquaculture feeds has focused on legumes such as beans, peas and lupins, as these plants are rich in digestible proteins and have favourable amino acid profiles [4]. While the soybean (*Glycine max*) from North and South America continues to dominate as a plant protein source for commercial and research aquaculture feeds, attempts are being made to diversify plant protein sources, with the emphasis on locally grown crops that would reduce the environmental impact of the fish farming industry [5].

Plants produce a wide range of secondary metabolites to defend themselves from predation by herbivores [6]. When consumed, these antinutritional factors (ANFs) typically interfere with digestion, absorption and/or utilisation of nutrients and have numerous adverse effects on animal health and performance [7]. Studies of animal transcriptome responses to ANFs have revealed that these effects are more pronounced when radical changes in the diet are made [8, 9] and in animals with no prior evolutionary experience to the plant materials and associated ANFs [10]. Indeed, feeding carnivorous fish like salmon with plant-based diets and exposing them to the evolutionary novel set of dietary toxins is one of the major challenges facing sustainable aquaculture, coupled with the need for each new plant ingredient to be evaluated for its potential health risks in a dose-dependent manner and in the context of potential interactions with other feed ingredients [11, 12].

Legumes are generally high in ANFs, but their specific profile varies from plant to plant and depends on the method used to extract proteins [13]. When salmon are fed diets containing full-fat or solvent extracted soybean meal (SBM), they develop an inflammatory condition in distal intestine, termed gut inflammation (enteritis), commonly characterized by shortening of mucosal folds, infiltration of the lamina propria by various inflammatory cells and decreased numbers of absorptive vacuoles in the enterocytes [14]. Further processing of SBM to produce soy protein concentrate (SPC) typically uses an aqueous alcohol wash, which removes alcohol-soluble ANFs, such as saponins. Since feeding salmon diets containing SPC does not induce major changes in gut histology or inflammatory responses that resemble the SBM-induced enteritis, development of this condition has been linked to the presence of saponins [15]. Recent studies with purified soy saponins supplemented to salmon diets containing FM, lupin kernel meal or pea protein concentrate (PPC) demonstrated that saponins do not necessarily cause enteritis on their own or without being potentiated by other ANFs [16–19]. Instead, they may act to increase gut permeability and therefore expose the local immune system to antigens that would not normally cross the intestinal epithelial barrier or would cross it at lower rates. Despite intensive research, the antigens responsible for triggering the SBM-induced inflammatory reaction in salmon gut have not yet been identified [20].

Further complexity is added by the fact that enteritis in salmon may also be induced by high dietary inclusions of plant materials that are relatively low in saponins. These include PPC made from the field pea (*Pisum sativum*) and bean protein concentrate (BPC) made from the faba bean (*Vicia faba*), both produced by fine grinding of dehulled seeds into flour, followed by air classification [5, 17]. It remains unknown whether enteritis induced by plant materials with notable differences in secondary metabolism has a common gene expression profile that generates altered gut histology or whether the mechanisms underlying the enteropathy are different (i.e., plant- or ANF-specific), but they subsequently converge at the level of altered gut histology as the inflammatory condition progresses.

Numerous approaches are available to investigate the relationship between dietary toxin exposure, toxicity and disease states [21]. Recent advances in molecular approaches enable the characterization of these effects at the cellular level, by studying the modulation of gene expression by bioactive substances in vivo. Given the number of saponins, proteinase inhibitors, lectins, oligosaccharides and other ANFs present in legumes, their potential interactions and dose-dependent effects on gut function in carnivorous fish are largely unknown. Furthermore, aquaculture feeds that utilise plant proteins are likely to affect multiple rather than individual genes and signalling pathways [22–24]. Global gene expression profiling studies are therefore required to provide insights into diet-gut interactions to ultimately aid protection of fish health in aquaculture.

In this study, we used Atlantic salmon parr to investigate transcriptome responses of distal gut to plant protein diets relative to a reference FM diet, following an 8-week feeding trial described in detail previously [5]. Fish were exposed to plant proteins with distinct sets of ANFs to establish whether similar or distinct alterations of the gut transcriptome were generated, and to characterise the gut transcriptome responses once the plant proteins were mixed. The single plant protein diets contained either SPC or BPC. SPC is typically low in the ANFs contributing to enteritis due to extensive processing [25], while BPC is characterised by high levels of condensed tannins and the presence of faba bean-specific glucosides such as vicine and convicine [26]. The mixed plant protein diets contained both SPC and BPC in three different proportions to generate graded inclusion levels of the plant materials under investigation. We also included an SBM diet to determine whether a distinction could be made between the gene expression profiles of gut inflammation induced by SBM and high levels of BPC. Finally, we investigated potential associations between the magnitude of changes in the gut transcriptome and aspects of fish performance, to provide insights into how diet-induced alterations of the gut transcriptome may affect the whole-animal physiology.

## Methods

### Diets

Ingredients and composition of the five experimental and two reference diets are described in detail elsewhere [5] and summarised in Table 1 and Additional file 1. The experimental diets were isonitrogenous, isolipidic and isocaloric and had similar contents of fish meal (~22 %) and similar total plant protein contents (~45 %), but differed in the proportions of soy protein concentrate (SPC) and bean protein concentrate (BPC). These two ingredients were used to replace each other (either partially or fully) at levels of incorporation ranging from 0 to 45 %. As a result, the experimental diets contained either 45 % SPC and 0 % BPC (S_45_), 34 % SPC and 11 % BPC (S_34_B_11_), 22 % SPC and 22 % BPC (S_22_B_22_), 11 % SPC and 34 % BPC (S_11_B_34_) or 0 % SPC and 45 % BPC (B_45_). The S_45_ and B_45_ diets were single plant protein diets, while the others (S_34_B_11_, S_22_B_22_ and S_11_B_34_) were mixed plant protein diets. The reference diets were enriched with fish meal (FM diet) or soybean meal (SBM diet) to provide negative and positive controls for gut inflammation, respectively. Diets S_45_, S_34_B_11_, S_22_B_22_, S_11_B_34_, B_45_, FM and SBM were the same as the previously described diets 20:80:00 (FM:SPC:BPC), 20:60:20, 20:40:40, 20:20:60, 20:00:80, FMref and HiSBM, respectively [5].

**Table 1:** Diet formulation, fish performance and histological evaluation of gut health

Parameter	Experimental plant protein diets					Reference diets	
	S_45_	S_34_B_11_	S_22_B_22_	S_11_B_34_	B_45_	FM	SBM
Key ingredient differences (g/100 g) between diets^1^							
Soy protein concentrate (SPC)	45	34	22	11	0	16	0
Bean protein concentrate (BPC)	0	11	22	34	45	0	0
Fish meal (FM)	22	22	22	22	22	56	44
Soybean meal (SBM)	0	0	0	0	0	0	36
Fish performance (growth rate and body composition)^2^							
Growth rate (g over 56 days)^3^	9.8^a^ (0.5)	9.7^a^ (0.3)	9.9^a^ (0.3)	10.1^a^ (0.2)	7.0^b^ (0.7)	13.1^c^ (0.2)	11.5^d^ (0.4)
Protein content (%)	16.2^ab^ (0.1)	16.5^c^ (0.1)	16.5^ac^ (0.2)	16.5^c^ (0.3)	16.1^b^ (0.2)	16.4^ac^ (0.1)	16.4^abc^ (0.1)
Lipid content (%)	9.6^a^ (0.2)	10.3^b^ (0.3)	10.0^ab^ (0.4)	10.2^b^ (0.4)	9.9^ab^ (0.1)	11.3^c^ (0.3)	11.0^c^ (0.4)
Ash content (%)	2.29^a^ (0.002)	2.25^bc^ (0.022)	2.26^b^ (0.023)	2.26^b^ (0.016)	2.27^ab^ (0.024)	2.22^d^ (0.017)	2.23^cd^ (0.013)
Histological scores from 1 (no enteritis) to 5 (severe enteritis) in distal gut^4^							
Sub-epithelial mucosa	1 (1–1)	1 (1–1)	1 (1–2)	1 (1–1)	1 (1–2)	1 (1–1)	1 (1–2)
Mucosal folds	2 (1–4)	2 (1–3)	3 (1–4)	2 (1–3)	3 (2–4)	3 (1–4)	3 (2–5)
Lamina propria	2 (1–2)	2 (1–3)	2 (1–3)	2 (1–3)	2 (1–2)	2 (1–3)	2 (2–3)
Eosinophilic granulocytes	2 (2–3)	2 (1–3)	2 (1–3)	2 (1–3)	2 (2–4)	2 (2–3)	2 (2–3)
Goblet cells^5^	3^a^ (1–4)	3^a^ (2–5)	3^a^ (2–4)	3^a^ (1–4)	3^b^ (2–4)	3^a^ (2–4)	3^b^ (2–4)
Supranuclear vacuoles^5^	2^a^ (1–5)	2^b^ (1–4)	2^a^ (1–4)	2^b^ (1–3)	4^c^ (2–5)	3^a^ (1–5)	5^c^ (3–5)
Gut health (based on histological scores)							
Enteritis presence and severity	-	-	-	-	mild	-	moderate

^1^See Additional file 1 for complete diet composition; ^2^values are means and standard deviations (in PARENTHESES), see [5] for details; ^3^different letters indicate significant differences between the diets (*P*-value < 0.05); ^4^values are medians and ranges (in PARENTHESES), see [5] for details; ^5^different letters indicate significant differences between the diets (*P*-value < 0.05), as assessed by multilevel ordinal regression [5]

### Animals and feeding trial

The study was conducted at the EWOS Innovation Research Facility (Dirdal, Norway), as described in detail elsewhere [5]. Briefly, Atlantic salmon of the SalmoBreed strain were supplied as fertilized eggs and hatched on site. Mixed-sex juvenile salmon in groups of ~150 fish were transferred to 26 indoor tanks (0.6 × 0.6 × 0.6 m), with ~60 L of freshwater flowing at a rate of 3.9 L/min and continuous aeration. The temperature of water was regulated at 13 °C and all animals were exposed to a constant light regime (24 h light/day). Fish were fed a commercial diet and acclimated to the experimental conditions for 2 weeks, after which (at body weight ~1.5 g) they were subjected to an 8-week feeding trial, consistent with other nutritional studies involving farmed salmon [22, 24, 27]. Tanks were randomly assigned to the dietary treatments, with four replicate tanks per each experimental diet (S_45_, S_34_B_11_, S_22_B_22_, S_11_B_34_ and B_45_) and three replicate tanks per each reference diet (FM and SBM diets). Fish were fed to satiation prior to and during the feeding trial using automatic band feeders (Holland Technology, Norway). During the feeding trial, all fish in each tank had their biomass recorded on days 0 (start of experiment), 14, 28, 42 and 56 (end of experiment) for evaluation of growth performance.

### Sampling

At the end of the feeding trial, fish from each tank were sampled for body composition and distal gut histology (as described in detail elsewhere [5]). For gene expression profiling of distal gut, five fish per tank for each experimental diet and seven fish per tank for each reference diet were killed and sampled. Prior to sampling, fish were starved for 24 h. For sampling, the peritoneal cavity was opened, alimentary tract excised and gut contents removed. The distal gut (defined as the region from the increase in intestinal diameter and presence of visible folds to the rectum) was subsequently cleaned of mesenteric and adipose tissue. Distal gut samples were immediately transferred to RNA*later*® (Sigma-Aldrich, St. Louis, MO, USA), kept at 4 °C overnight and then stored at −80 °C prior to RNA extraction.

### Statistical analysis of fish performance

The effects of diets on growth rate and protein, lipid and ash contents were determined using one-way ANOVA. Fisher post hoc pairwise comparisons were used to compare individual treatment contrasts where required. Modelling assumptions were assessed using standard plots of residuals. All analysis was performed using Minitab 16 (Minitab Inc., State College, PA, USA) and statistical significance was determined at *P*-value < 0.05.

### RNA isolation

Total RNA from distal gut was isolated by homogenization of ~100 mg of tissue in TRIzol® Reagent (Ambion by Life Technologies, Carlsbad, CA, USA), using 3 mm tungsten carbide beads and a TissueLyser II Disruption System (Qiagen GmbH, Hilden, Germany). Following isolation, the RNA was quantified by spectrophotometry (NanoDrop Technologies, Wilmington, DE, USA), had integrity confirmed by electrophoresis (Agilent Technologies, Santa Clara, CA, USA) and was then stored at − 80 °C for later processing.

### Design of microarray experiment, RNA amplification and labelling

The individual RNA samples were pooled to generate five biological replicates per each dietary treatment, with an equimolar contribution of RNA from four fish to each pool (~10 μg of RNA per fish per pool). Fish from the same pool were either from four replicate tanks (experimental diets; one fish per tank) or from three replicate tanks (reference diets; 1-2 fish per tank, with one randomly selected tank contributing twice to the same pool). As a result, each dietary treatment was represented by RNA samples from 20 fish (5 RNA pools × 4 fish per pool). All 35 experimental RNA pools (7 diets × 5 biological replicates) were subsampled to generate a common control, with contribution of RNA samples from 140 fish (7 diets × 20 fish per diet). Antisense amplified RNA (aRNA) with amino allyl UTP incorporation was generated from ~2 μg total RNA per experimental or common control pool, using the Amino Allyl MessageAmp™ II aRNA Amplification Kit (Ambion by Life Technologies, Carlsbad, CA, USA), as described previously [28].

Pools of aRNA were subjected to a coupling reaction between the amino allyl-modified UTP residues and amine reactive Cy fluorescent dyes (Amersham™ Cy™3 and Cy™5 Mono-reactive Dye Packs; GE Healthcare UK Limited, Little Chalfont, UK), as described previously [29]. All experimental samples were labelled with Cy3, and Cy5 was used to label the common control. The reaction products were purified using a DyeEx® 2.0 Spin Kit (Qiagen GmbH, Hilden, Germany). Dye incorporation and post-labelling aRNA yield were quantified by spectrophotometry (NanoDrop Technologies, Wilmington, DE, USA).

### Microarray hybridisation, scanning and feature extraction

The current experiment used a custom designed Agilent oligonucleotide microarray platform Salar_2 (Agilent design ID: 025520), developed for Atlantic salmon and validated by two independent research groups using RT-qPCR [24, 28–30]. The experiment consisted of 35 hybridisations (7 diets × 5 biological replicates), which were performed on nine microarray slides in a semi-randomised order (each slide with four different dietary treatments).

Each hybridisation was performed using 825 ng of Cy3-labelled experimental sample and 825 ng of Cy5-labelled common control. The aRNA was first fragmented and then hybridised at 65 °C for 17 h in an Aligent hybridization oven, as described previously [29]. Following hybridisation, slides were subjected to washing steps, after which they were air dried in the dark and scanned within 2 h.

Scanning was carried out at 5 μm resolution on a GenePix Personal 4100A scanner (Axon Instruments, Molecular Devices Corp., Sunnyvale, CA, USA), with the PMT values adjusted manually to ensure the mean intensity ratio of Cy3:Cy5 signal was close to one. Agilent Feature Extraction Software (version 9.5.3) was used to identify features and extract raw intensity values associated with these features for subsequent statistical analysis.

### Microarray data analysis

Feature intensities were pre-processed and differential gene expression between dietary treatments identified using R [31] and the Bioconductor package limma [32]. Briefly, loess normalisation was performed within each array to account for intensity-dependent variation in dye bias and quantile normalisation was used to stabilize experimental variances across arrays. Normalised data were then filtered to remove control features and, additionally, non-responsive RNA targets with equal expression across treatments were removed by non-specific filtering [33]. Differential expression of RNA targets between diets was assessed using linear modelling and empirical Bayes methods [34]. Comparisons between diet groups were made by extracting specific contrasts from the linear model. These contrasts were set up to compare each plant protein diet with the reference FM diet. Multiple testing was accounted for by controlling the false discovery rate (FDR) at 5 % using the Benjamini-Hochberg procedure. The RNA targets with adjusted *P-*value < 0.2 and absolute fold change ≥ 1.4 were considered as differentially expressed. The relatively low stringency of the adopted cut-off criteria is consistent with other nutrigenomic studies [35]. All RNA targets meeting these criteria (*n* = 1558) are listed in Additional file 2.

### Gene annotation and functional analysis

The salmon RNA targets were mapped to human orthologs to generate HGNC (HUGO Gene Nomenclature Committee) gene symbols for downstream functional analysis. This approach has been demonstrated to improve functional analysis of fish genes by providing access to well-annotated databases and tools for mammalian model organisms, despite limitations of the mapping due to the extra genome duplication events in teleost fish and species differences in gene function and pathways [36]. Each salmon sequence was annotated with the best hit in the human proteome from the Ensembl database (BLASTX), but only hits with *E*-value < 0.001 were accepted. As a result, 1341 of 1558 (86 %) differentially expressed RNA targets were annotated, with a number of redundant RNA targets that were mapped to the same gene. The fold changes of the redundant features were then averaged to provide a single fold change for each gene. A total number of 1058 differentially expressed genes (Additional file 2) were identified for subsequent functional analysis.

Sets of differentially up-regulated and down-regulated genes (140, 80, 33, 71, 254 and 750 genes for S_45_, S_34_B_11_, S_22_B_22_, S_11_B_34_, B_45_ and SBM dietary treatments, respectively) were analysed using IPA (Ingenuity Pathway Analysis, QIAGEN Redwood City, www.qiagen.com/ingenuity) to identify pathways that were significantly altered by dietary manipulation at *P*-value (calculated by Fisher exact test right-tailed) < 0.01. These pathways are reported in Additional file 3, along with their ratios (number of differentially regulated genes divided by the total number of genes within a specific pathway in the IPA reference gene set, called the Ingenuity® Knowledge Base) and IPA activation z-scores (an algorithm to predict the direction of change for a given pathway, with z-score ≥ 2 suggesting an overall increase in the pathway activity and z-score ≤ −2 predicting an overall decrease in the pathway activity). Furthermore, the IPA downstream effects analysis was performed to identify biological functions that were expected to increase or decrease, taken into account genes’ direction of change, i.e., up-regulated or down-regulated status of the differentially expressed genes. This analysis is based on expected causal effects between genes and functions derived from the literature compiled in the Ingenuity® Knowledge Base, and uses the z-score algorithm to make predictions whether the function is significantly increased (z-score ≥ 2), significantly decreased (z-score ≤ 2), non-significantly modified (−2 < z-score < 2) or with no clear pattern of activation related to the literature (no z-score calculated).

The same sets of differentially expressed genes were also submitted to DAVID Bioinformatics Resources 6.7 [37, 38] to examine the enrichment of Gene Ontology (GO) terms for biological processes against the human reference gene set. The analysis was performed on category GOTERM_BP_FAT using the functional annotation tool with a default threshold of 2 counts. The GO terms with a modified Fisher exact *P-*value (EASE score) < 0.01 were considered significantly enriched (Additional file 4).

## Results

### Phenotypic characteristics of fish

**Fig. 1:**
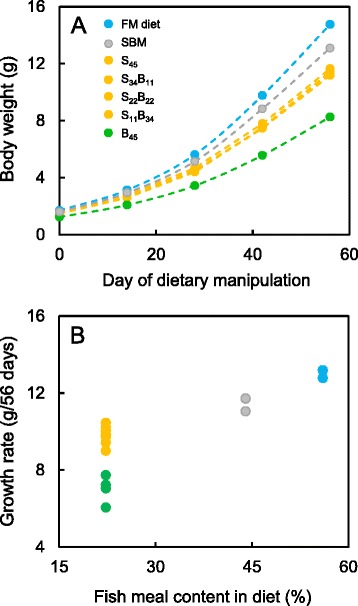
Body weight and growth of fish during an 8-week feeding trial. Different colours indicate significant differences in growth rate of fish between diets (*P*-value < 0.05, see Table 1 for details). Dietary manipulations were performed on four replicate tanks for each experimental diet (S_45_, S_34_B_11_, S_22_B_22_, S_11_B_34_ and B_45_) and three replicate tanks for reference FM and SBM diets. Body weight data for each tank were generated by recording the biomass of all (~150) fish in the tank, divided by the number of fish. Growth rate of fish for each tank was calculated as the initial body weight (day 0) subtracted from the final body weight (day 56). **a** Body weight of fish calculated for each diet as the mean across replicate tanks (error bars are omitted for simplicity). **b** Growth rate of fish for each replicate tank plotted against the fish meal content of the diet

Changes in the phenotype of the fish subjected to the dietary manipulation are described in detail elsewhere [5] and summarised in Table 1. Briefly, salmon with higher content of fish meal in the diet grew faster, while salmon fed the same level of fish meal but different levels of soy and bean protein concentrates had similar growth rates, apart from the group fed the diet containing 45 % BPC (B_45_), which grew significantly slower (Fig. 1). High levels of fish meal in the reference FM and SBM diets (56 and 44 %, respectively) had no effect on salmon protein content, but fish fed these diets tended to have higher lipid and lower ash contents than fish fed the plant protein diets with 22 % of fish meal in the diet. Furthermore, fish fed the mixed plant protein diets (S_34_B_11_, S_22_B_22_ and S_11_B_34_) had significantly higher protein (16.5 % *vs* 16.1–16.2 %) and lipid (10.0–10.3 % *vs* 9.6–9.9 %) contents and lower ash contents (2.25–2.26 % *vs* 2.27–2.29 %) compared with fish fed the single plant protein diets (S_45_ and B_45_).

Histological examination of distal gut in fish fed experimental and reference diets involved scoring six histological parameters (sub-epithelial mucosa, mucosal folds, lamina propria, eosinophilic granulocytes, goblet cells and supranuclear vacuoles) from 1 (no enteritis) to 5 (severe enteritis). Neither sub-epithelial mucosa, mucosal folds, lamina propria nor eosinophilic granulocytes were affected by dietary manipulation. In contrast, both goblet cells and supranuclear vacuoles had elevated scores for enteritis in fish fed B_45_ and SBM diets. Overall, fish fed the FM diet (a negative control for enteritis) as well as fish fed S_45_, S_34_B_11_, S_22_B_22_ and S_11_B_34_ diets had no signs of gut inflammation, fish fed B_45_ diet showed signs of mild enteritis, while fish on the SBM diet (a positive control for enteritis) developed moderate enteritis.

### Magnitude of gut transcriptome responses to single *vs* mixed plant protein diets

Among all seven diets used in this study, the reference FM diet most closely resembled the natural diet of salmon. We therefore considered the distal gut of fish fed the FM diet as being characterised by gene expression typical for a healthy digestive system. All responses of gut transcriptome to the plant protein diets were expressed relative to the FM diet, with up-regulation and down-regulation referring to the higher and lower levels of gene expression in fish fed plant protein diets than in fish fed the FM diet, respectively.

**Fig. 2:**
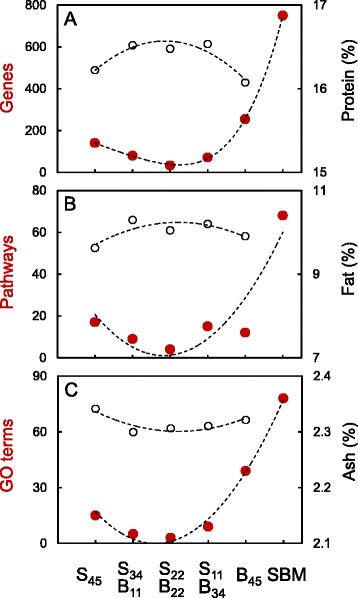
Magnitude of gut transcriptome responses to dietary plant proteins. **a** Number of differentially expressed genes (adjusted *P*-value < 0.2, absolute fold change ≥ 1.4) for single (S_45_ and B_45_) *vs* mixed (S_34_B_11_, S_22_B_22_ and S_11_B_34_) plant protein diets and SBM diet (positive control for enteritis), relative to FM diet (negative control for enteritis). **b** Number of enriched pathways (*P*-value < 0.01) identified by Ingenuity Pathway Analysis (IPA). **c** Number of enriched Gene Ontology (GO) terms for biological processes (*P*-value < 0.01), identified by DAVID functional annotation tool. Aspects of fish performance (mean values of body protein (**a**), fat (**b**) and ash (**c**) content) are also shown

Single plant protein diets with either 45 % SPC (S_45_) or 45 % BPC (B_45_) induced more extensive changes in gut transcriptome than mixed plant protein diets (S_34_B_11_, S_22_B_22_ and S_11_B_34_), based on the number of differentially expressed genes (Fig. 2a). Specifically, the S_45_ and B_45_ diets were associated with changes in 140 and 254 genes, respectively, whereas only 80, 33 and 71 genes were altered by S_34_B_11_, S_22_B_22_ and S_11_B_34_ diets, respectively (Additional file 2). Similar trends with more extensive changes in the gut transcriptome induced by single plant protein diets compared with mixed plant protein diets were also observed for the numbers of enriched pathways (Fig. 2b, Additional file 3) and the numbers of enriched GO terms for biological processes (Fig. 2c, Additional file 4). Importantly, fish fed the mixed plant protein diets (with smaller alterations of gut transcriptome) had higher protein and lipid contents and lower ash content than fish fed either S_45_ or B_45_ diets (with larger alterations of gut transcriptome) (Table 1, Fig. 2). Overall, the most extensive changes in gut transcriptome (alterations of 750 genes, 68 pathways and 78 GO terms) were found in the fish fed the SBM diet that induced moderate enteritis (details below).

Analysis of the common and unique features altered in the gut transcriptome by single and mixed plant protein diets revealed no obvious synergistic interactions between soy and bean protein concentrates (Additional file 5). Instead, many features (at gene, pathway and GO term levels) altered in the gut of fish fed mixed plant protein diets (S_34_B_11_, S_22_B_22_ and S_11_B_34_) were also altered by single plant protein (S_45_ and B_45_) or SBM diets. The focus of subsequent analysis was therefore to characterise the gut transcriptome responses to the diets with single plant protein ingredients, either highly processed (SPC), moderately processed (SBM) or air-classified (BPC), and determine whether the observed effects persisted, disappeared or existed at the dose-dependent intermediate levels after the plant proteins were mixed.

### Specificity of gut transcriptome responses to soy and bean proteins

**Fig. 3:**
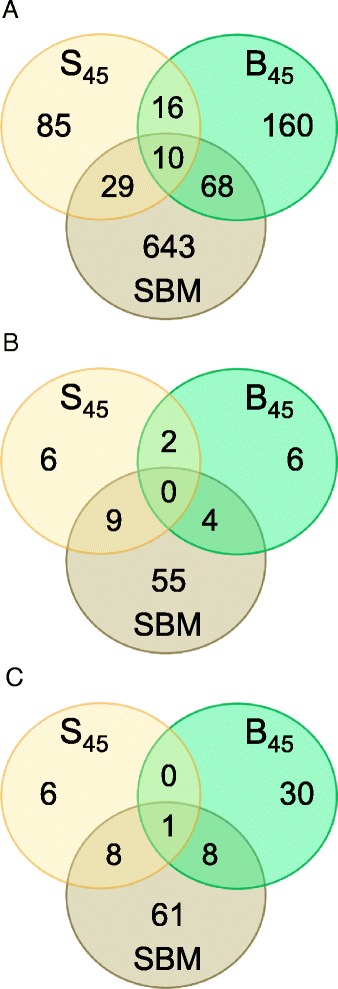
Characterisation of gut transcriptome responses to dietary plant proteins. Venn diagrams show the numbers of common and unique features altered in gut transcriptome by single plant protein diets, enriched with either soy protein concentrate (S_45_), bean protein concentrate (B_45_) or soybean meal (SBM). **a** Genes differentially expressed (adjusted *P*-value < 0.2, absolute fold change ≥ 1.4) in the gut of fish fed the single plant protein diets, relative to FM diet. **b** Enriched pathways (*P*-value < 0.01) identified by Ingenuity Pathway Analysis (IPA). **c** Enriched Gene Ontology (GO) terms for biological processes (*P*-value < 0.01), identified by DAVID functional annotation tool

Analysis of common and unique features altered in the distal gut of fish fed diets containing 45 % SPC (S_45_), 45 % BPC (B_45_) or 36 % SBM revealed that all these ingredients generated highly specific gut transcriptome responses, reflecting the differences in the origin of the plant protein (soy *vs* bean) as well as differences in the level of processing protein from the same plant (highly processed SPC *vs* moderately processed SBM) (Fig. 3, Additional files 6, 7 and 8). The S_45_ diet was associated with significant alterations of 140 genes, 17 pathways and 15 GO terms, of which changes in 85 genes, six pathways and six GO terms were S_45_-specific. The majority of non-specific responses to this diet were shared with the SBM treatment rather than with the B_45_ diet, indicating the presence of the common signature of the soy protein that was not affected by the extraction method used to produce SPC. Among 254 genes, 12 pathways and 39 GO terms altered by the B_45_ diet, 160 genes, six pathways and 30 GO terms were B_45_-specific. The B_45_ diet shared more alterations with the SBM diet rather than with the S_45_ diet, suggesting the common signature of gut inflammation induced by both these diets. Finally, the gut transcriptome responses to the SBM diet was also highly specific, with 643 of 750 genes, 55 of 68 pathways and 61 of 78 GO terms being unique for that treatment.

### Effects of diet with 45 % of soy protein concentrate (S_45_)

Among 17 pathways significantly altered in the distal gut of fish fed S_45_ diet, cholesterol biosynthesis pathway (details below) was the most enriched (Additional file 9). Consistent with these changes were also changes mapped to biosynthesis of zymosterol, an intermediate in the biosynthesis of cholesterol. Furthermore, the long-chain polyunsaturated fatty acid (LC-PUFA) biosynthesis pathway and the oleate biosynthesis pathway were also highly enriched.

The S_45_ diet had relatively little impact on gut inflammatory and immune responses, as only one pathway (complement system) related to these functions was affected. The alteration of this pathway was unique for the S_45_ diet and associated with the altered expression of CFD, SERPING1 and C7 genes, all of which were down-regulated. Other pathways specific for the S_45_ diet included mismatch repair in eukaryotes, hypoxia signalling in the cardiovascular system and PCP pathways.

Neither differential expression of genes encoding the cytochrome P450 superfamily of enzymes (CYP51A1, CYP2F1 and CYP2A7) nor pathways associated with these genes were specific to the S_45_ diet. These pathways included bupropion degradation, acetone degradation, estrogen biosynthesis, nicotine degradation and melatonin degradation, and their significant enrichments were shared either with the B_45_ or SBM diets.

The IPA downstream effects analysis predicted significant changes in the gut of fish fed the S_45_ diet, related to two functional categories (Table 2, Additional file 10). The first was an increase in synthesis of steroid, based on the expression profile of 8 genes (ACAT2, CFTR, CREB1, CYP51A1, DHCR7, FDFT1, G6PC and SLC27A2). The second predicted change was a decrease in concentration of triacylglycerol, with nine genes involved (ALOX5, CACNA1B, FABP6, FADS2, G6PC, JUN, MGAT3, PRF1 and TXNIP).

**Table 2:** IPA downstream effects analysis performed on genes differentially expressed in distal gut of Atlantic salmon

Diet/Function	Predicted activation state	Activation z-score	*P*-value	Number of genes^a^		
				consistent^b^	inconsistent^c^	unknown^d^
S_45_ (diet with 45 % of soy protein concentrate)						
synthesis of steroid	Increased	2.0	2.2E-03	4	0	4
concentration of triacylglycerol	Decreased	−2.4	1.1E-03	8	1	0
B_45_ (diet with 45 % of bean protein concentrate)						
cell-cell adhesion	Decreased	−2.6	8.0E-07	7	0	7
growth of epithelial tissue	Decreased	−2.4	3.2E-05	17	7	2
development of blood vessel	Decreased	−2.1	3.5E-05	16	6	4
cell movement of epithelial cells	Decreased	−2.0	2.7E-05	8	2	0
cell death of smooth muscle cells	Decreased	−2.1	1.3E-03	6	1	0
apoptosis of connective tissue cells	Decreased	−2.1	3.3E-03	8	2	1
Soybean meal (SBM) diet						
proliferation of cells	Increased	2.4	4.7E-15	125	90	52
binding of DNA	Increased	2.3	1.5E-05	28	14	6
synthesis of protein	Increased	2.1	6.6E-07	22	10	12
synthesis of lipid	Increased	2.4	1.6E-06	30	15	20
metabolism of carbohydrate	Increased	2.6	1.5E-04	16	4	30
organization of cytoplasm	Increased	2.3	2.4E-04	38	21	38
cellular homeostasis	Increased	2.4	2.0E-05	43	27	37
cell movement	Increased	3.4	1.1E-07	87	47	15
cell death	Decreased	−2.1	6.5E-14	124	88	36
autophagy	Increased	2.5	1.6E-04	18	6	6

^a^Genes are listed in Additional file 10; ^b^Genes with the direction of expression consistent with the predicted activation state of the function (e.g., gene encoding CD28 molecule is known to decrease cell proliferation and is down-regulated in the SBM dataset, therefore predicted to increase the function); ^c^Genes with the direction of expression inconsistent with the predicted activation state of the function (e.g., gene encoding DIABLO is known to decrease cell proliferation and is up-regulated in the SBM dataset, therefore predicted to decrease the function); ^d^Genes involved in the function with currently unknown effects

### Effects of diet with 45 % of bean protein concentrate (B_45_)

Among 12 pathways altered in the gut transcriptome of fish fed high inclusion of BPC, seven of these pathways were associated with differential expression of three genes encoding integrins, the cell adhesion molecules (Additional file 11). Two of these genes (ITGA6 and ITGB1) were B_45_-specific, while ITGB3 was also differentially expressed in the distal gut of fish fed the SBM diet. Among other genes that substantially contributed to the gut signature of the high-bean diet were TNF, a multifunctional proinflammatory cytokine, and its receptor TNFRSF1A.

**Fig. 4:**
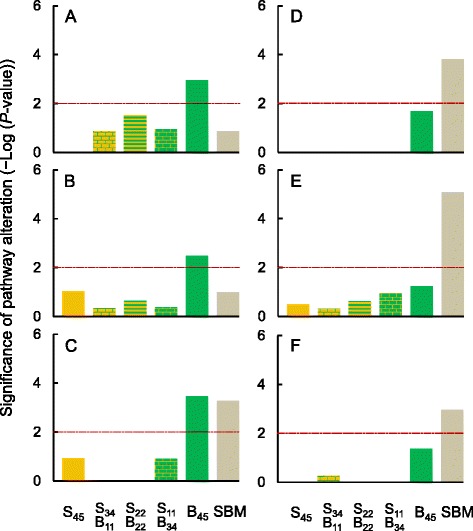
Top pathways altered in distal gut of Atlantic salmon. **a**, **b** and **c** Pathways altered by the diet with 45 % of bean protein concentrate (B_45_) included **a** granulocyte adhesion and diapedesis, **b** tight junction signalling and **c** ILK signalling. **d**, **e** and **f** Pathways altered by soybean meal diet (SBM) included **d** production of nitric oxide and reactive oxygen species in macrophages and **e** NRF2-mediated oxidative stress response and **e** actin cytoskeleton signalling. The pathways and their significance were identified by Ingenuity Pathway Analysis (IPA). The cut-off point for pathway significance (*P*-value < 0.01, equal to –Log_10_ (*P*-value) > 2) is depicted as the dotted horizontal line

Consistent with the altered gut histology (mild enteritis), the B_45_ diet triggered in the gut transcriptome a number of inflammatory and immune responses that were either B_45_-specific or common for the B_45_ and SBM diets. In total, six pathways were specific to the high-bean diet, including granulocyte adhesion and diapedesis (Fig. 4a) and agranulocyte adhesion and diapedesis (no graphic representation). Both these pathways are involved in the migration of granulocytes (neutrophils, basophils and eosinophils) and agranulocytes (lymphocytes and monocytes) from the vascular system to sites of pathogenic exposure and are the key events in the process of inflammation. Highly enriched and specific to the high-bean diet was also caveolar-mediated endocytosis signalling pathway, important for endocytic internalization of a variety of particles (including molecules, viruses and bacteria) and acting through caveolae rather than clathrin lattices. Finally, the significant alterations of the tight junction signalling pathway (Fig. 4b) suggested the potential of B_45_ diet to modify the gut epithelial permeability, especially via a paracellular route, for which tight junctions are the main regulators.

The gut transcriptome responses common for the B_45_ and SBM diets included alteration of ILK (integrin-linked kinase) signalling pathway (Fig. 4c), implicated in cellular responses to a wide variety of extracellular stimuli. Other aspects of the common signature of gut inflammation induced by these diets resembled the alterations of signalling between the junction of germ cells and Sertoli cells for timely germ cell movement within the seminiferous epithelium (germ cell-Sertoli cell junction signalling pathway). Furthermore, both diets induced gut transcriptome changes related to clathrin-mediated endocytosis signalling pathway, with clathrin-mediated endocytosis being the major pathway for the internalization of nutrients, hormones and other signalling molecules from the plasma membrane into intracellular compartments.

According to the IPA downstream effects analysis, the high-bean diet affected many aspects of intestinal epithelial development and function (Table 2, Additional file 10). Among the most affected were decreased cell-cell adhesion and impaired growth of epithelial tissue, coupled with reduced development of blood vessel and decreased cell movement of epithelial cells. The prediction was also made with regard to cell death of smooth muscle cells and apoptosis of connective tissue cells, with both functions decreased.

### Effects of soybean meal (SBM) diet

As expected, the SBM diet altered gut histology (moderate enteritis) and induced extensive changes in gut transcriptome, represented in this study by alterations of 750 genes, 68 pathways and 78 GO terms (Fig. 3). These changes are important to understand the potential mechanisms underlying SBM-induced enteropathy and provide insights into a coordinated series of molecular, cellular, tissue and systemic responses that aim to control inflammation and restore gut homeostasis and function.

In total, 20 of 68 pathways were associated with various aspects of inflammatory and immune responses (both innate and acquired), 18 of which were unique to the SBM treatment (Additional file 12). Despite a relatively long exposure to the SBM diet (8 weeks), some aspects of gut transcriptome responses were typical for the early defence systems, such as acute phase response, NF-κB and IL-8 signalling pathways, known to stimulate the synthesis of various proinflammatory cytokines and mediators. These alterations may suggest the excessive translocation of luminal bacteria, viruses and antigens across the intestinal epithelium, which was further supported by the enrichment of the pathways related to endocytosis, such as clathrin-mediated endocytosis signalling (shared with B_45_ diet), macropinocytosis signalling and virus entry via endocytic pathways. The potential contribution of microbial translocation to SBM-induced enteritis was reinforced by the substantial up-regulation of the IFNG transcript and the enrichment of associated pathways (e.g., role of PKR in interferon induction and antiviral response) as well as modification of pathways directly linked to the intestinal barrier function and permeability (e.g., remodeling of epithelial adherens junctions and epithelial adherens junction signalling). Furthermore, our transcriptomic data were consistent with the increased phagocytic activity of macrophages (Fcγ receptor-mediated phagocytosis in macrophages and monocytes pathway) and neutrophils (fMLP signalling in neutrophils pathway), coupled with the highly significant alteration of the pathway related to production of nitric oxide and reactive oxygen species in macrophages (Fig. 4d). Finally, the SBM-induced enteritis was associated with the transcriptomic activation of T cells by alteration of pathways related to CD28 signalling and CTLA4 signalling, as well as modification of the T cell receptor signalling pathway. According to our gene expression data, some of these T cells may have potentially responded to activation by killing other cells via the induction of a programmed cell death known as apoptosis (enrichment of the pathway related to cytotoxic T lymphocyte-mediated apoptosis of target cells).

The distal gut of fish fed the SBM diet showed at the transcriptomic level clear signs of increased cellular damage and injury. Part of this damage was likely to result from the increased production of nitric oxide and reactive oxygen species, known for their microbicidal and tumoricidal properties and generated by phagocytic cells to control infection, but inevitably affecting also the host cells. Indeed, the pathways associated with the detoxification of reactive intermediates and alleviation of oxidative stress (e.g., NRF2-mediated oxidative stress response and eNOS signalling) were highly enriched in the gut transcriptome of fish fed the SBM diet (Fig. 4e). The effects of the SBM diet on gut gene expression were also consistent with the accumulation of unfolded and misfolded proteins in endoplasmic reticulum (ER) lumen, as suggested by the alteration of ER stress pathway. The presence of the ER stress was further supported by the transcriptomic activation of unfolded protein response pathway, indicating the attempts to resolve the protein folding defect and restore ER homeostasis or promoting apoptosis (under unresolvable ER stress conditions). In fact, some aspects of the SBM-induced ER stress were probably unresolvable, because the inflamed gut tissue generated (at the transcriptomic level) multiple signals known to initiate apoptosis, with the up-regulation of DIABLO and CASP8 genes among the most important. The pathways associated with the apoptosis signalling (e.g., Myc-mediated apoptosis signalling, apoptosis signalling and death receptor signalling) were also highly enriched.

The changes induced by the SBM diet in the gut transcriptome were consistent with increased cellular proliferation and migration. These changes included the up-regulation of PCNA (a universal marker of proliferating cells) as well as alterations of the pathways associated with cell cycle control, such as cell cycle: G2/M DNA damage checkpoint regulation. An increased migration of cells within the inflamed gut tissue was supported by the enrichment of pathways related to actin cytoskeleton signalling and integrin signalling, essential for cytoskeletal remodelling during cell adhesion, loss of attachment and successive re-adhesion of migrating cells (Fig. 4f).

Increased cell proliferation of the intestinal tissue with the SBM-induced enteritis was also predicted by the IPA downstream effects analysis, along with concomitant increases in binding of DNA, synthesis of protein, synthesis of lipid, metabolism of carbohydrate, organization of cytoplasm, cellular homeostasis and cell movement (Table 2, Additional file 10). The prediction was also made with regard to increased autophagy. Despite the activation of apoptosis signalling pathways, cell death in the inflamed gut tissue was predicted to decrease.

### Dose-dependent alterations of cholesterol and LC-PUFA biosynthesis pathways

Dose effects of dietary plant proteins on distal gut gene expression were established by examining the relationship between SPC/BPC content and the significance of pathway alteration (−Log_10_ (*P*-value)). The effects of SPC could not be separated from the effects of BPC, because both ingredients were changing simultaneously to replace each other.

**Fig. 5:**
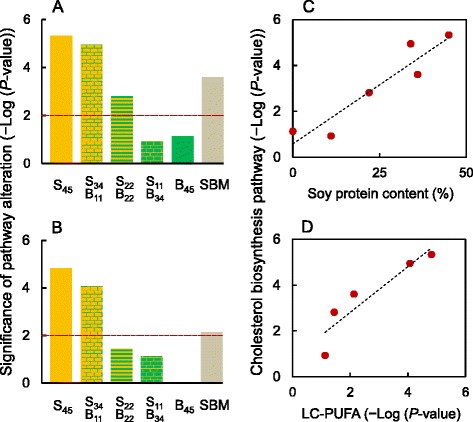
Transcriptomic alterations of cholesterol and LC-PUFA biosynthesis pathways in distal gut of Atlantic salmon. **a** and **b** Significance of pathway alteration (−Log_10_ (*P*-value)) for **a** cholesterol biosynthesis and **b** LC-PUFA biosynthesis pathway in relation to diets S_45_, S_34_B_11_, S_22_B_22_, S_11_B_34_ and SBM, containing 45 % of soy protein concentrate (SPC), 34 % SPC and 11 % of bean protein concentrate (BPC), 22 % SPC and 22 % BPC, 11 % SPC and 34 % BPC, and soybean meal (SBM), respectively. The pathways and their significance were identified by Ingenuity Pathway Analysis (IPA). The cut-off point for pathway significance (*P*-value < 0.01, equal to –Log_10_ (*P*-value) > 2) is depicted as the dotted horizontal line. **c** and **d** The significance of pathway alteration for cholesterol biosynthesis plotted against **c** soy protein content in the diet and **d** the significance of pathway alteration for LC-PUFA biosynthesis pathway. Both associations are significant (*P*-value < 0.05) and fitted with the lines that represent reduced major axis regressions

The dose effects of SPC/BPC were most clearly visible in the cholesterol biosynthesis pathway, with the highest levels of alteration in the gut of fish fed S_45_ and S_34_B_11_ diets, the lowest levels when fish were fed S_11_B_34_ and B_45_ diets, and intermediate levels for S_22_B_22_ and SBM diets (Fig. 5a). Correlations between the significance of the pathway alteration and the highly or moderately processed soy protein contents (*r* = 0.94, *P*-value = 0.006, *n* = 6 diets, Fig. 5c) as well as the BPC content (*r* = −0.88, *P*-value = 0.020, *n* = 6 diets, no graphic representation) were both highly significant. In total, 7 of 39 genes mapped to the cholesterol biosynthesis pathway were significantly altered by our dietary manipulations (Table 3). All these genes were up-regulated relative to the FM diet, which may suggest an overall increase in the pathway activity.

**Table 3:** Transcriptomic alterations of cholesterol and LC-PUFA biosynthesis pathways in distal gut of Atlantic salmon

Pathway/Gene	Gene fold change relative to fish meal (FM) diet^a^					
	S_45_	S_34_B_11_	S_22_B_22_	S_11_B_34_	B_45_	SBM
Cholesterol biosynthesis						
FDFT1, farnesyl-diphosphate farnesyltransferase 1	1.8					2.9
CYP51A1, cytochrome P450, family 51, subfamily A, polypeptide 1	3.1	2.3				2.6
TM7SF2, transmembrane 7 superfamily member 2	2.1	1.8	1.7			1.5
MSMO1, methylsterol monooxygenase 1					2.5	2.4
NSDHL, NAD(P) dependent steroid dehydrogenase-like						2.4
SC5D, sterol-C5-desaturase	2.5	1.9				1.9
DHCR7, 7-dehydrocholesterol reductase	1.9	1.9	2.2	1.9	1.6	1.6
LC-PUFA biosynthesis						
ACSBG2, acyl-CoA synthetase bubblegum family member 2				15.3		
ACSL5, acyl-CoA synthetase long-chain family member 5						−4.2
ACSL6, acyl-CoA synthetase long-chain family member 6						−1.6
CYB5A, cytochrome b5 type A (microsomal)						1.7
CYB5R3, cytochrome b5 reductase 3	1.6					1.5
FADS2, fatty acid desaturase 2	2.1	2.0	2.4			
SLC27A2, solute carrier family 27 (fatty acid transporter), member 2	1.7	1.6				

S_45_, S_34_B_11_, S_22_B_22_, S_11_B_34_, B_45_ and SBM are diets with 45 % of soy protein concentrate (SPC), 34 % SPC and 11 % of bean protein concentrate (BPC), 22 % SPC and 22 % BPC, 11 % SPC and 34 % BPC, 45 % BPC, and enriched with soybean meal (SBM), respectively

^a^Only genes considered as differentially expressed (adjusted *P-*value < 0.2, absolute fold change ≥ 1.4) are reported

The dose-dependent alteration profile of the LC-PUFA biosynthesis pathway (annotated as γ-linolenate biosynthesis pathway) (Fig. 5b) closely resembled the profile of cholesterol biosynthesis pathway (Fig. 5a), despite different sets of genes involved in these pathways (Table 3). In total, seven of 24 genes mapped to the LC-PUFA biosynthesis pathway were altered, including up-regulation of the FADS2 gene, known to catalyse biosynthesis of 18:4n-3 from 18:3n-3 and 18:3n-6 from 18:2n-6. The alteration levels of LC-PUFA and cholesterol biosynthesis pathways were highly correlated (*r* = 0.92, *P*-value = 0.025, *n* = 5 diets, Fig. 5d).

## Discussion

The present study is the first to demonstrate that mixing plant proteins with distinct sets of ANFs induces less extensive changes in the gut transcriptome of carnivorous fish than using diets with a single plant protein source. In our investigation, we assumed that the gut of fish fed the reference FM diet was characterised by levels of gene expression typical of a healthy digestive system. At the level of the transcriptome, the guts from fish on diets containing both SPC and BPC were closer to that healthy baseline than the guts from fish on diets with either SPC or BPC (Fig. 2). Further support for this assumption comes from the changes in gut transcriptome induced by plant proteins relative to the FM diet, where there was a good agreement between the transcriptomic and histological data. Specifically, the transcriptomic changes were the greatest in the distal gut showing histological signs of moderate enteritis (SBM diet), intermediate in the gut showing mild enteritis (B_45_ diet), and the smallest in the guts having no histological signs of gut inflammation (S_45_, S_34_B_11_, S_22_B_22_ and S_11_B_34_ diets). Despite the gene expression profile and histological changes consistent with gut inflammation, fish fed the SBM diet grew significantly faster than fish with healthy guts on the experimental diets (Table 1, Fig. 1). This result may be explained by the high levels of FM in the SBM diet (44 % *vs* 22 % in the experimental diets), which are known to promote growth of carnivorous fish and are commonly added in excess to mitigate the inflammatory condition induced by SBM during nutritional experiments [39]. In contrast, mild enteritis was likely implicated in the reduced growth rate of fish fed the B_45_ diet, which contained the standard levels of FM (22 %).

The primary aim of the present study was to determine whether plant proteins with distinct sets of ANFs produced similar or different changes in the gut transcriptome of carnivorous fish. We generated gene expression profiles corresponding to three diets with either 45 % SPC (S_45_), 45 % BPC (B_45_) or SBM, and demonstrated that these diets evoked profoundly different alterations of the gut transcriptome, with a relatively small number of features (genes, pathways and GO terms) that were altered commonly by any two or all three treatments (Fig. 3). Importantly, the two soy-based diets shared more pathways with each other (*n* = 9) than with the bean-based diet (two pathways common for S_45_ and B_45_ diets, and four pathways common for SBM and B_45_ diets). Furthermore, the pathways shared by the soy-based diets had typically similar sets of genes altered, while the pathways common for the soy- and bean-based diets had usually different sets of genes affected by the dietary manipulations (Additional files 9, 11 and 12), supporting our findings about the ANF-specific alterations of gut transcriptome in Atlantic salmon. Overall, our results are consistent with the use of microarray technology to distinguish between gene expression patterns generated by structurally unrelated toxicants in the livers of laboratory rats [21], natural and novel plant secondary compounds in the livers of desert woodrats [10] and acute and chronic mycobacterial infections in zebrafish [40].

Since the gut transcriptome alterations induced by SPC and BPC were either dose-dependent (e.g., cholesterol and LC-PUFA biosynthesis pathways), associated with the high dose of SPC (e.g., complement system pathway) or associated with the high dose of BPC (e.g., granulocyte adhesion and diapedesis pathway), mixing those two plant protein ingredients reduced the absolute amount of each of them in the diet, which subsequently resulted in reduced transcriptomic changes in the gut (Fig. 2). It is important to note that even the plant proteins considered as most suitable for use in aquaculture and showing no associated histological abnormalities in the gut, such as SPC, altered the gut transcriptome when used at high doses (45 % in this study). Hence, elimination of those high dose effects by diversification of plant protein ingredients used for aquaculture feeds may be important for controlling individual and cumulative levels of ANFs to which fish are exposed, leading to improvements in fish health and performance. Indeed, fish fed the mixed plant protein diets with relatively small alterations of gut transcriptome were characterised by higher protein and lipid contents and lower ash content than fish fed diets with either 45 % SPC or 45 % BPC (Fig. 2). The causality of this relationship was not addressed in our study and requires further investigation.

Both SBM and B_45_ diets significantly altered the histology of the distal gut and induced moderate and mild gut inflammation, respectively (Table 1). These diets had distinct sets of ANFs, which in turn generated profoundly different alterations of gut transcriptome, with some aspects of transcriptomic modifications common for both diets. The nature of these common responses (especially alterations of ILK signalling and germ cell-Sertoli cell junction signalling pathways) suggests that although the mechanisms by which different ANFs affect gut health may be different, they are all likely to contribute to the overall loss of intestinal integrity, thus facilitating antigenic translocation and promoting the persistence of enteritis. We believe that the overlapping gut transcriptomic responses to SBM and B_45_ diets are of special interest for aquaculture as they may harbour biomarkers that characterise all types of gut inflammatory diseases, independent of their origin and aetiology. Identification of these potential biomarkers and their further validation is beyond the scope of this study.

## Conclusions

In summary, we have demonstrated that different plant protein materials, such as SPC and SBM made from soybean and BPC made from faba bean, generated substantially different gene expression profiles in the distal gut of Atlantic salmon relative to guts from fish fed a FM-based diet, with relatively few transcriptomic alterations common for all plant proteins used. These different expression patterns likely reflect different sets of ANFs present in the highly (SPC) and moderately (SBM) processed soybean and air-classified faba bean (BPC). When different plant proteins (SPC and BPC) were mixed, they generated less extensive alterations of the gut transcriptome relative to single plant protein diets with either SPC or BPC, probably due to reduced levels of individual ANFs. The mixed plant protein diets were also associated with an improved body composition of the fish relative to the effects seen with single plant protein diets, which may provide evidence for a link between the magnitude of changes in the gut transcriptome and whole-animal performance. Furthermore, fish with more advanced gut inflammation (moderate enteritis induced by the SBM diet, assessed histologically) had more extensive alterations of gut transcriptome than fish with mild enteritis, induced by the high levels of BPC in the diet. The common aspects of these predominantly different gene expression profiles of SBM- and BPC-induced enteritis may have additional diagnostic value when fish are screened for gut health and potential enteropathies. Our results clearly indicate that gut transcriptomic profiling provides a useful tool for testing the applicability of alternative protein sources for aquaculture feeds and for designing diets that have a reduced impact of ANFs on fish health.

## Perspectives and significance

Our paper addresses the complex diet-gut interactions and intestinal homeostasis that are of special interest for the fish farming industry for a number of reasons [41]. Firstly, farmed fish kept at high stocking densities are susceptible to intestinal infections, with the gut being an important entry point for pathogens. Secondly, farmed fish are typically fed commercial pelleted feeds, which opens up avenues for manipulating fish health through the incorporation of various drugs and vaccines into the feed. Thirdly, the gut immune system of teleost fish allows microbial colonization by symbionts, and this microbial community is a potential platform to modulate fish pathogens. Finally, gut microbiota in fish are likely to respond to dietary manipulations [42]. Hence, a comprehensive understanding of the diet-gut interactions and immunoregulatory properties of intestinal epithelium in fish could aid in the development of new strategies to prevent and treat their multiple infectious and inflammatory diseases. Ultimately, understanding the diet-gut interactions and intestinal homeostasis in farmed fish is important to maximise performance and to ensure that aquaculture continues to be a sustainable source of food for the growing world population.

Attempts to understand the diet-gut interactions are important because of their widespread ramifications across many areas of inquiry beyond aquaculture. The gastrointestinal tract of vertebrates along with its single layer of epithelial cells constitutes the largest and most important barrier against the external environment [43]. The intestinal epithelium acts as a selectively permeable barrier for dietary nutrients, electrolytes and water, while maintaining an effective defence against luminal toxins, antigens and microbial communities. The epithelial cells are also crucial mediators of mucosal innate and adaptive immunity, and they actively contribute to maintaining intestinal homeostasis [44]. The latter may be at risk, when humans and other animals consume evolutionary ‘mismatched’ diets, i.e., diets they did not evolve to digest, absorb and utilise, and which typically come with novel sets of dietary toxins, antigens and microbial challenges. These factors are likely to contribute to the increased intestinal epithelial permeability, which has been implicated in the predisposition to intestinal inflammation and a number of gastrointestinal diseases in humans [43], zoo animals [45] and pets [46], laboratory rodents [47], domesticated livestock [48] and poultry [49]. Evidence is also growing that many wild animals are facing new dietary challenges resulting from global climate change [50, 51]. Our results clearly indicate that farmed Atlantic salmon may provide an attractive new animal model for investigating the complex interactions between the digestive system and evolutionary mismatched diets in vertebrates, at both whole-animal and molecular levels.

### Availability of supporting data

The data sets supporting the results of this article are available in the ArrayExpress repository (http://www.ebi.ac.uk/arrayexpress/) under accession numbers A-MEXP-2065 (Salar_2 array design) and E-MTAB-3613 (microarray raw data).

### Ethical statement

The feeding trial and sampling procedures were approved by the Ethical Committee of the Institute of Aquaculture (University of Stirling, UK) and performed in compliance with laws regulating experimentation with live animals in Norway, as overseen by the Norwegian Animal Research Authority (Forsøksdyrutvalget). All diets used in this trial met the nutritional requirements of the Atlantic salmon.

## Additional files

Additional file 1:
**Complete ingredient and chemical composition of experimental and reference diets.** (PDF 186 kb) Additional file 2:
**List of RNA targets differentially expressed in distal gut of Atlantic salmon fed plant protein diets, relative to control fish meal (FM) diet, with** **cut-off** **criteria of adjusted** ***P-*** **value < 0.2 and absolute Log**_**2**_ **fold change ≥ 0.5 (equal to absolute fold change ≥ 1.4).** Plant-protein diets were enriched with either soy protein concentrate (SPC), bean protein concentrate (BPC), both SPC and BPC or soybean meal (SBM). Fish RNA targets were mapped to human orthologs (HGNC gene names and symbols) using Ensembl database (BLASTX, *E*-value < 0.001). The fold changes of the redundant RNA targets were averaged to provide a gene fold change. (XLSX 335 kb) Additional file 3:
**List of pathways significantly altered (** ***P*** **-value < 0.01) in distal gut of Atlantic salmon fed plant protein diets.** The Ingenuity Pathway Analysis (IPA) was performed on sets of 140, 80, 33, 71, 254 and 750 differentially expressed genes (both up- and down-regulated) for S_45_, S_34_B_11_, S_22_B_22_, S_11_B_34_, B_45_ and SBM dietary treatments, respectively. Ratios are numbers of differentially regulated genes divided by the total numbers of genes within a specific pathway that are in the IPA reference gene set. The IPA activation z-score ≥ 2 predicts an overall increase in the pathway activity and z-score ≤ −2 predicts an overall decrease in the pathway activity. (XLSX 25 kb) Additional file 4:
**List of Gene Ontology (GO) terms for biological processes that were significantly altered (** ***P*** **-value < 0.01) in distal gut of Atlantic salmon fed plant protein diets.** The analysis was performed using DAVID functional annotation tool, on sets of 140, 80, 33, 71, 254 and 750 differentially expressed genes (both up- and down-regulated) for S_45_, S_34_B_11_, S_22_B_22_, S_11_B_34_, B_45_ and SBM dietary treatments, respectively. (XLSX 42 kb) Additional file 5:
**Venn diagrams showing the numbers of common and unique features altered in distal gut of Atlantic salmon by single (S**_**45**_ **, B**_**45**_ **and SBM) and mixed (S**_**34**_ **B**_**11**_ **, S**_**22**_ **B**_**22**_ **and S**_**11**_ **B**_**34**_ **) plant protein diets.** (PDF 73 kb) Additional file 6:
**List of common and unique genes that were significantly altered (adjusted** ***P*** **value < 0.2, absolute fold change ≥ 1.4) in distal gut of Atlantic salmon by single plant protein diets, enriched with either soy protein concentrate (S**_**45**_ **), bean protein concentrate (B**_**45**_ **) or soybean meal (SBM), relative to fish meal (FM) diet.** (XLSX 83 kb) Additional file 7:
**List of common and unique pathways that were significantly altered (** ***P*** **-value < 0.01) in distal gut of Atlantic salmon by single plant protein diets, enriched with either soy protein concentrate (S**_**45**_ **), bean protein concentrate (B**_**45**_ **) or soybean meal (SBM).** (XLSX 13 kb) Additional file 8:
**List of common and unique Gene Ontology (GO) terms for biological processes that were significantly altered (** ***P*** **-value < 0.01) in distal gut of Atlantic salmon by single plant protein diets, enriched with either soy protein concentrate (S**_**45**_ **), bean protein concentrate (B**_45_ **) or soybean meal (SBM).** (XLSX 16 kb) Additional file 9:
**Pathways (** ***n*** **= 17) and contributing genes altered in distal gut of Atlantic salmon fed a diet with 45 % of soy protein concentrate (S**_**45**_ **).** (XLSX 27 kb) Additional file 10:
**List of genes associated with biological functions predicted by IPA downstream effects analysis to increase or decrease in distal gut of Atlantic salmon fed single plant protein diets.** (XLSX 14 kb) Additional file 11:
**Pathways (** ***n*** **=** **12) and contributing genes altered in distal gut of Atlantic salmon fed a diet with 45 % of bean protein concentrate (B**_**45**_ **).** (XLSX 26 kb) Additional file 12:
**Pathways (** ***n*** **= 68) and contributing genes altered in distal gut of Atlantic salmon fed a soybean meal (SBM) diet.** (XLSX 79 kb)
