# Supplementary material for: Differential responses of the gut transcriptome to plant protein diets in farmed Atlantic salmon
Source: BMC Genomics. 2016 Feb 29;17:156. doi: 10.1186/s12864-016-2473-0 (PMC4772681; doi:10.1186/s12864-016-2473-0)
Supplement: Additional file 1: — Complete ingredient and chemical composition of experimental and reference diets. (PDF 186 kb) [file 12864_2016_2473_MOESM1_ESM.pdf]

Table 1 Complete ingredient and chemical composition of experimental and reference diets

Parameter	Experimental plant protein diets[1]					Reference diets[2]	
	S_{45}	$S_{34}B_{11}$	$S_{22}B_{22}$	$S_{11}B_{34}$	B_{45}	FM	SBM
Ingredients (g/100 g)[3,4,5]							
Soy protein concentrate (SPC)	44.82	33.62	22.41	11.21	0	16.00	0
Bean protein concentrate (BPC)[6]	0	11.18	22.36	33.54	44.72	0	0
Fish meal (FM)[7]	22.24	22.24	22.24	22.24	22.24	56.00	44.00
Soybean meal	0	0	0	0	0	0	36.00
Wheat gluten	8.00	8.00	8.00	8.00	8.00	8.00	0
Methionine	0.31	0.35	0.39	0.43	0.47	0	0
Lysine	0.52	0.52	0.52	0.52	0.52	0	0
Threonine	0	0.04	0.08	0.12	0.16	0	0
Fish oil	9.84	9.76	9.68	9.61	9.53	9.00	9.00
Nobacithin	1.00	1.00	1.00	1.00	1.00	1.00	1.00
Tapioca	7.61	7.63	7.66	7.67	7.70	8.00	8.00
Vitamin mix[8]	0.17	0.17	0.17	0.17	0.17	0.17	0.17
Vitamin C	0.10	0.10	0.10	0.10	0.10	0.10	0.10
Mineral mix	0.10	0.10	0.10	0.10	0.10	0.10	0.10
Monocalcium phosphate	5.16	5.16	5.16	5.16	5.16	1.50	1.50
Magnesium sulphate	0.05	0.05	0.05	0.05	0.05	0.05	0.05
Lucantin pink	0.08	0.08	0.08	0.08	0.08	0.08	0.08
Total	100	100	100	100	100	100	100
Analysed chemical composition (g/100 g)[3,5]							
Protein	51.8	50.0	51.5	51.0	51.8	54.5	48.9

Lipid	12.1	10.9	11.1	11.1	11.4	15.2	12.8
Starch	7.8	8.0	8.1	7.4	6.3	6.9	7.2
Ash	9.4	9.4	9.5	9.6	9.6	8.6	8.9
Moisture	7.4	9.6	8.0	8.4	7.5	8.7	10.7

[1] Diets S_{45}, $S_{34}B_{11}$, $S_{22}B_{22}$, $S_{11}B_{34}$ and B_{45} are the same as the previously described diets 20:80:00 (FM:SPC:BPC), 20:60:20, 20:40:40, 20:20:60, 20:00:80, respectively [1]; [2] Diets FM and SBM are the same as the previously described diets FMref and HiSBM, respectively [1]; [3] wet mass; [4] all ingredients sourced from EWOS stocks, unless otherwise stated; [5] see [1] for details; [6] Fabaqua 62-65 (Sotexpro, Berméricourt, France); [7] Norse-LT 94 (Egersund Fisk AS, Norway); [8] no vitamin C included.

References

1. De Santis C, Ruohonen K, Tocher DR, Martin SAM, Król E, Secombes CJ, Bell JG, El-Mowafi A, Crampton VO. Atlantic salmon (*Salmo salar*) parr as a model to predict the optimum inclusion of air classified faba bean protein concentrate in feeds for seawater salmon. Aquaculture. 2015;444:70-78.
