# Supplementary material for: Differential responses of the gut transcriptome to plant protein diets in farmed Atlantic salmon
Source: BMC Genomics. 2016 Feb 29;17:156. doi: 10.1186/s12864-016-2473-0 (PMC4772681; doi:10.1186/s12864-016-2473-0)
Supplement: Additional file 5: — Venn diagrams showing the numbers of common and unique features altered in distal gut of Atlantic salmon by single (S 45 , B 45 and SBM) and mixed (S 34 B 11 , S 22 B 22 and S 11 B 34 ) plant protein diets. (PDF 73 kb) [file 12864_2016_2473_MOESM5_ESM.pdf]

Additional file 5

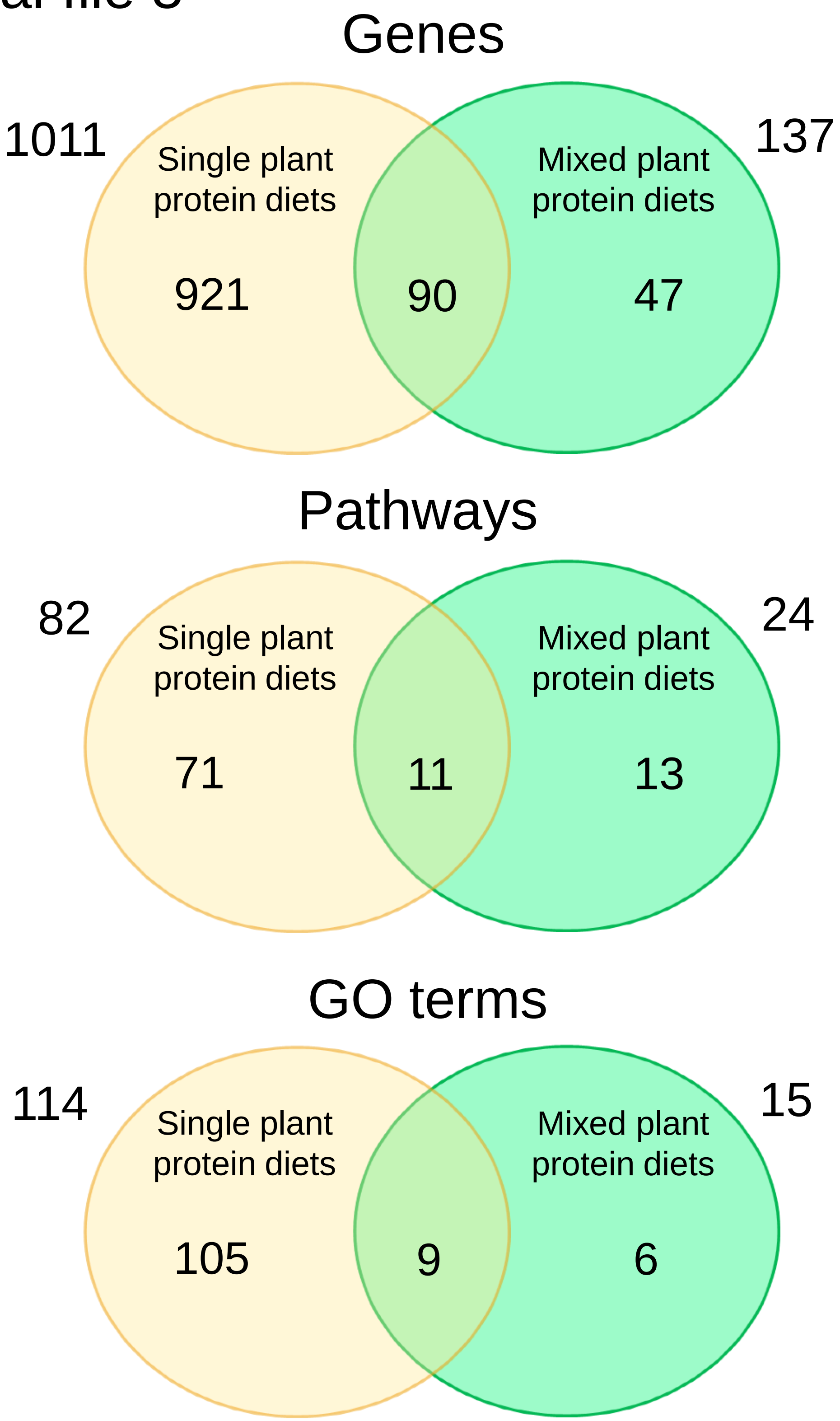

Venn diagrams showing the numbers of common and unique features altered in gut transcriptome by single (S_{45}, B_{45} and SBM) and mixed ($S_{34}B_{11}$, $S_{22}B_{22}$ and $S_{11}B_{34}$) plant protein diets
